# The Relationship Between Nursing Students' Perception of Obstetric Violence, Pre-Pregnancy Fear of Childbirth, and Moral Sensitivity: A Cross-Sectional Study

**DOI:** 10.1155/da/4843962

**Published:** 2025-07-24

**Authors:** Nülüfer Erbil, Özlem Akın Yamak, Hilal Gül Boyraz Yanık

**Affiliations:** ^1^Department of Obstetrics and Gynecologic Nursing, Faculty of Health Sciences, Ordu University, Ordu, Türkiye; ^2^Department of Obstetrics and Gynecologic Nursing, Faculty of Health Sciences, Recep Tayyip Erdoğan University, Rize, Türkiye

**Keywords:** childbirth fear, moral sensitivity, nursing students, obstetric violence

## Abstract

**Objective:** Nursing students' perceptions of obstetric violence and moral sensitivity can affect their pre-pregnancy fear of childbirth levels. This study was conducted to examine the relationship between nursing students' moral sensitivity, perception of obstetric violence, and pre-pregnancy fear of childbirth.

**Methods:** The research was designed as a cross-sectional study. This study was conducted between November 1, 2024, and November 15, 2024, with the participation of 315 nursing students. Data were collected face-to-face using the Personal Information Form, Pre-pregnancy Fear of Childbirth Scale, Obstetric Violence Perception Scale, and Moral Sensitivity Questionnaire (MSQ). Ethical committee approval and institutional permission were obtained before starting the study. Parametric data were analyzed using *t*-tests and one-way ANOVA, while nonparametric tests were analyzed using the Mann–Whitney *U* test and Kruskal–Wallis test. Pearson correlation analysis and linear regression analysis were also conducted. Statistical significance was set at *p*  < 0.05.

**Results:** The average age of the students included in the study was 21.67 ± 1.37, with 50.5% being 4th-year students, 79.7% being female, and 66% choosing nursing as their profession voluntarily. It was determined that 66% of the participants preferred vaginal birth, 38.1% had previously heard the term obstetric violence, 18.1% had witnessed someone being subjected to obstetric violence, and 89.2% thought that nurses or midwives needed communication knowledge and skills. A weak correlation was found between the Pre-pregnancy Fear of Childbirth Scale and the Obstetric Violence Perception Scale (*r* = 0.134, *p*=0.018). It was also determined that the predictor of pre-pregnancy fear of childbirth was the students' perception of obstetric violence (*R* = 0.170, *R*^2^ = 0.028, *p*=0.013).

**Conclusion:** The study concluded that the perception of obstetric violence is a predictor of pre-pregnancy fear of childbirth. Additionally, a negative and weak relationship was found between students' moral sensitivity scores and ‘intrapartum mistreatment.

## 1. Introduction

Obstetric violence is any harm inflicted on women during pregnancy, childbirth, and the postpartum period, encompassing disrespectful and abusive treatment, physical and verbal abuse, failure to provide necessary care, withholding medications, and coercive or nonconsensual medical interventions [1]. The prevalence of obstetric violence has been found to vary between 15% and 91%, depending on the country, the tools and methods used, the definition and types of obstetric violence, and the culture surrounding childbirth [2–4]. In a study conducted in Türkiye, it was reported as 76.4% [5]. Despite the increasing prevalence of behaviors associated with obstetric violence, there is no global consensus on which behaviors can be defined as violence or how to asure this phenomenon, making it difficult to determine the exact prevalence of obstetric violence [6].

Pregnancy, childbirth, and motherhood are among the unforgettable experiences in a woman's life. Being subjected to disrespectful or abusive care can lead to childbirth being perceived as traumatic by women, leaving lasting negative effects [7, 8]. In particular, negative birth experiences accompanied by anxiety are thought to play a significant role in the development of childbirth fear, which may lead to an increase in cesarean section rates and a decline in pregnancy rates in the future [9]. On the other hand, interventions provided by midwives have been shown to reduce cesarean birth rates and increase the preference for vaginal delivery in future pregnancies [10, 11].

It is stated that nursing students' witnessing of obstetric violence during the birth process affects their perception of birth [12]. It has been found that negative experiences, especially in clinical practice, are an important factor affecting fear of birth [13].

The existence and perpetuation of obstetric violence reveal the lack of education for health professionals [14]. In this context, since the attitudes and behaviors of health professionals toward violence related to birth are shaped in the early stages of their education, it is important to create a critical awareness of obstetric violence at the undergraduate level [15]. Because nursing students' perception of obstetric violence and fear of birth are critical for their professional development and the quality of care they will provide in the future [12].

Nurses play a crucial role in alleviating pregnant women's fears about childbirth and providing positive birth support during the preparation phase [16]. During the caregiving process, an interaction based on moral values emerges between the nurse and the healthy or ill individual. In this process, making the right decisions and adopting ethical values guide nurses in their professional practice within a moral framework and help them establish an ethical connection with patients [17–19]. Studies have found that a lack of moral sensitivity can cause nurses to overlook common ethical issues in daily practice, leading to irrational clinical decisions and nurse–patient conflicts [20, 21].

Professional values can be a guide in practice in nursing care [22, 23]. Therefore, by acquiring professional and ethical values and integrating them into their professional development, nursing students will make the care they provide more respectful and qualified [24]. In addition, the moral sensitivity of nursing students will ensure that they perform their professions with ethical responsibility, thus contributing to providing patient-centered, respectful, and compassionate care.

Based on these studies, it is believed that nursing students' perceptions of obstetric violence and their moral sensitivity may influence their levels of childbirth fear before pregnancy. It is believed that this study will contribute to filling the existing gap in the literature regarding the topic. In this regard, the findings obtained from the planned study could help determine nursing students' perceptions of obstetric violence and their moral sensitivity, enabling the planning of training sessions for healthcare professionals, both actively working and in training. Additionally, interventions targeting childbirth fears could be developed. This study was conducted to examine the relationship between nursing students' moral sensitivity, perceptions of obstetric violence, and pre-pregnancy childbirth fears.

### 1.1. Research Questions

• What are the levels of moral sensitivity among nursing students?
• What are nursing students' perceptions of obstetric violence?
• What are nursing students' levels of pre-pregnancy childbirth fear?
• What are the factors affecting nursing students' pre-pregnancy childbirth fears?

## 2. Method

### 2.1. Type of Research

The research was designed as a cross-sectional study.

### 2.2. Population and Sample of the Study

The population of the study consisted of 229 students from the 3rd and 4th-year Nursing Department at Ordu University Faculty of Health Sciences and 209 students from the 3rd and 4th-year Nursing Department at Recep Tayyip Erdoğan University Faculty of Health Sciences, totaling 438 students. The sample of the study includes a total of 315 nursing students from the 3rd and 4th years of the Nursing Departments at two state universities in the Eastern Black Sea Region of Turkey. The students who volunteered to participate in the study and agreed to participate in the study reached 72% (71.91%) of the students. In the universities where the study was conducted, the women's health and diseases nursing course is taken in the 3rd year. Since students' perceptions of pregnancy and birth are strengthened during this process and they have the potential to have experience and awareness of obstetric violence, 3rd and 4th year nursing students were included in the study, while 1st and 2nd years were not included in the study because they had not yet taken theoretical and practical courses.

### 2.3. Inclusion Criteria for the Study

Nursing students who are 18 years of age or older, have taken theoretical and practical courses in women's health and diseases nursing, and have agreed to participate in the study were included.

### 2.4. Exclusion Criteria for the Study

Students who initially agreed to participate but later decided to withdraw at any stage of the study were excluded.

### 2.5. Data Collection Method and Duration

The research data were collected between November 1, 2024, and November 15, 2024, through face-to-face interviews after obtaining informed consent from the students. The forms and scales were filled out by the participants themselves. The data collection process took ~10–15 min.

### 2.6. Data Collection Tools

#### 2.6.1. Personal Information Form

The “Personal Information Form,” created by the researchers based on the literature, includes questions that describe characteristics such as age, class, marital status, chronic illness, and mental health conditions of the students [25–27].

#### 2.6.2. Childbirth Fear-Prior to Pregnancy Scale (CF-PPS)

Developed by Stoll et al. [28], this scale measures pre-pregnancy childbirth fear among young women and men and is a self-report tool. The validity and reliability study was conducted by Uçar and Taşhan [29]. The Likert-type scale consists of 10 items, with responses ranging from “strongly disagree” (1) to “strongly agree” (6). A score between 10 and 60 can be obtained from the scale, with higher scores indicating a higher level of fear. In the original study, the Cronbach's alpha value was determined to be 0.86 [29]. In this study, the Cronbach's alpha value was calculated as 0.893.

#### 2.6.3. Questionnaire for Measuring Perceived Obstetric Violence (PercOV-S)

The “Questionnaire for Measuring Perceived Obstetric Violence” was developed by Mena-Tudela et al. [14] to assess perceptions of obstetric violence. The Turkish validity and reliability study was conducted by Gönenç et al. [30]. The scale consists of 27 items and five subscales. The subscales are “intrapartum mistreatment,” “non-evidence-based routine practices,” “postpartum mistreatment,” “accompaniment and consent,” and “intervention without indications.” The minimum possible score on the scale is 27, and the maximum score is 135. There are no reverse-coded items on the scale. The statements in the scale reflect the respondent's opinion on whether a situation is perceived as obstetric violence or not, with responses ranging from “Definitely Not Obstetric Violence,” “Mostly Not Obstetric Violence,” “Partially Obstetric Violence,” “Mostly Obstetric Violence,” to “Definitely Obstetric Violence.” An increase in the total score on the scale indicates a higher perception and awareness of obstetric violence. The Cronbach's alpha reliability coefficients for the scale were found to be 0.93, with subscale values ranging from 0.69 to 0.90 [30]. In this study, the Cronbach's alpha value was found to be 0.844.

#### 2.6.4. Moral Sensitivity Questionnaire (MSQ)

The MSQ was developed by Lutzen et al. [31] to measure ethical sensitivity. It is a seven-point Likert-type scale consisting of 30 items and six subscales. The subscales of the MSQ include Autonomy, Beneficence, Holistic Approach, Conflict, Application, and Orientation. The minimum possible score on the MSQ is 30, and the maximum score is 210. A low score indicates high ethical sensitivity, while a high score indicates low ethical sensitivity. The Turkish validity and reliability study was conducted by Tosun [32], with a Cronbach's alpha value of 0.84. In this study, the Cronbach's alpha value was calculated as 0.845.

### 2.7. Ethical Aspects of the Study

Permission to use the CF-PPS, Perceived Obstetric Violence Scale, and the MSQ was obtained by email from the authors. Ethical approval for the study was granted by the Non-Interventional Clinical Research Ethics Committee of Recep Tayyip Erdoğan University (decision number 2024/126), and written institutional permissions were obtained from the rectorships and faculty deans of the universities to carry out the research. The study adhered to the principles of the Helsinki Declaration. Informed written consent was obtained from the students who volunteered to participate in the study.

### 2.8. Data Analysis

Data analysis was performed using statistical software. Descriptive statistical methods, including frequency, percentage, mean, standard deviation, minimum and maximum values, and parametric and nonparametric tests, were used to evaluate the data. The normality of the data distribution was assessed using Skewness and Kurtosis coefficients. For normally distributed data, differences between independent groups were evaluated using *t*-tests and one-way ANOVA. For nonnormally distributed data, differences between independent groups were assessed using the Mann–Whitney *U* test and Kruskal–Wallis test. Correlations between continuous variables were tested using Pearson correlation analysis, and cause-and-effect relationships between variables were tested with linear regression analysis. Cronbach's alpha coefficients were used for the reliability analysis of the scales. Statistical significance was set at *p*  < 0.05.

## 3. Results

The average age of the 315 students included in the study was 21.67 ± 1.37, with 50.5% being 4th-year students and 79.7% being female. It was determined that 66% of the students chose the nursing profession voluntarily. Among the students, 30.8% had previously experienced vaginal birth, 52.4% reported hearing negative birth stories, and 30.2% preferred cesarean delivery for themselves or their partner in the future. Furthermore, 66% of the participants preferred vaginal delivery for themselves, while 38.1% had heard the term “obstetric violence” before, and 18.1% had witnessed someone experiencing obstetric violence. It was found that 89.2% of the students believed that nurses or midwives needed better communication skills. Additionally, 29.5% of students did not think healthcare professionals were ethically responsible during the birth process, while 53% reported that their views on childbirth, their partner's birth, or marriage had changed based on observed births or services provided to women's health, and 45.7% of their opinions had been negatively affected (Table 1).

The students' average score on the CF-PPS (43.60 ± 9.17) was found to be above the medium level. The students' average score on the PercOV-S (89.16 ± 13.24) was also above the medium level, while the average score on the MSQ (93.13 ± 18.68) was found to be below the medium level (Table 2).

Among the female students in the study, the average CF-PPS score was found to be higher than that of the male students, with a statistically significant difference (*p*  < 0.05, Table 1). Those who preferred cesarean delivery had higher CF-PPS scores compared to those preferring vaginal delivery. Similarly, students who had witnessed women experiencing obstetric violence had higher CF-PPS scores than those who had not, and the differences were statistically significant (*p*  < 0.05). Students who did not believe healthcare professionals were ethically responsible during the birth process had higher CF-PPS scores compared to those who did, with statistically significant differences (*p*  < 0.05, Table 1). Furthermore, students who reported that observed births or healthcare services influenced their views on childbirth, their partner's childbirth, or marriage had higher CF-PPS scores than those who did not, with statistically significant differences (*p*  < 0.05, Table 1). No statistically significant differences in CF-PPS scores were found based on other characteristics of the students (*p*  > 0.05, Table 1).

The study found that female students had significantly higher average PercOV-S scores compared to male students, and those who had vaginal deliveries had higher PercOV-S scores compared to those who had cesarean deliveries (*p*  < 0.05, Table 1). No statistically significant differences were found in PercOV-S scores based on other characteristics of the students (*p*  > 0.05, Table 1). Similarly, no statistically significant differences were observed in MSQ scores based on students' characteristics (*p*  > 0.05, Table 1).

A weak positive correlation was found between the CF-PPS scores and PercOV-S scores (*r* = 0.134, *p*=0.018). There was also a weak correlation between CF-PPS scores and the “intrapartum mistreatment” (*r* = 0.154) and “ routine practices without evidence ” (*r* = 0.126) subscales of the PercOV-S (*p*  < 0.05, Table 3). Furthermore, there was a weak negative correlation between MSQ scores and the “intrapartum mistreatment” subscale of PercOV-S (*r* = −0.143, *p*  < 0.05).

The study identified that obstetric violence perception was a predictor of pre-pregnancy fear of childbirth (*R* = 0.170, *R*^2^ = 0.028, *p* =0.013), as shown in Table 4.

## 4. Discussion

Pregnancy and childbirth are multidimensional processes that evoke various emotions. These emotions can be experienced not only by pregnant women and their partners but also by women and men who have not yet had children [33]. In this study, the average CF-PPS score of students was found to be above the medium level (Table 2). A study with university students found that one in four women reported clinically significant levels of fear [27]. In studies conducted in Europe, North America, and Australia, about 26%–27% of nonpregnant female students reported high levels of childbirth fear [27, 34, 35].

Research on childbirth fear has mostly focused on pregnant and postpartum women. However, attitudes toward childbirth can be shaped before the first pregnancy. Therefore, it has been noted that investigations into the prevalence of fear in young women who have not yet conceived, and how such fears are formed through persistent tendencies or information from various sources, are needed [27]. Additionally, in this study, it was found that female students had higher and statistically significant birth fear scores compared to male students, which is consistent with other studies indicating that women generally have higher levels of childbirth fear than men [36, 37]. This can be attributed to women directly experiencing pregnancy and childbirth processes.

Childbirth fear may be a common reason for unnecessary cesarean deliveries [11]. The literature reports that women in various cultures often request planned cesareans due to fear of childbirth [38–40]. In this study, it was found that those who preferred cesarean delivery had higher and statistically significant CF-PPS scores compared to those who preferred vaginal delivery (Table 1). Supporting this finding, Stoll et al. [41] observed that university students with higher levels of childbirth fear were more likely to opt for cesarean delivery.

In this study, it was found that 38.1% of students had heard the term obstetric violence before, and 18.1% had witnessed someone experiencing obstetric violence (Table 1). In studies conducted in India and the United Kingdom, despite having completed their obstetrics and gynecology rotations, medical students reported that they had never encountered the term obstetric violence during their education [26]. While 14% of medical students in the United Kingdom reported seeing examples of obstetric violence during their clinical practice, 49% of students in India reported witnessing it [26]. The insufficient and superficial treatment of this issue during medical education has been suggested as a reason for students' lack of knowledge on the subject [42]. To address and prevent obstetric violence, practical training on professional behaviors and communication skills can be provided to all healthcare professionals.

Another finding of this study was that students who had witnessed women experiencing obstetric violence had higher and statistically significant CF-PPS scores compared to those who had not witnessed such events (Table 1). To address this issue, raising awareness about obstetric violence among health sciences students during their initial training is essential [14]. A study with health sciences students showed that educational interventions on obstetric violence during their initial training could change their perceptions of obstetric violence, and it was also found that obstetric violence could negatively affect students' fear of childbirth [43]. In this study, a weak correlation was found between CF-PPS scores and PercOV-S scores (Table 3). Similarly, previous studies have shown a significant positive relationship between traumatic birth perceptions and fear of childbirth, indicating that students who witnessed traumatic events had higher levels of these perceptions and fears [44, 45]. Understanding how nursing students' perceptions of obstetric violence and childbirth fear are related suggests that improving nurses' positive perceptions, as they play a central role in patient communication, could lead to better outcomes in patient care.

It is important for future nursing professionals to recognize different forms of obstetric violence and be familiar with this issue, as they are expected to provide comprehensive and humane care during every stage of the birth process [15, 42]. In this study, it was found that the students' MSQ scores were below average, indicating high ethical sensitivity (Table 2). A study by Arries [46] showed that nursing students' professional value levels influenced their ethical ideologies. Previous studies have found that clinical nurses with higher ethical decision-making skills are more successful in establishing good nurse-patient relationships, providing high-quality care, and avoiding ethical conflicts [47–49]. In this study, 29.5% of students did not believe that healthcare professionals were aware of their moral responsibilities during the birth process (Table 1). Furthermore, it was found that students who did not think healthcare professionals were aware of their moral responsibilities had significantly higher CF-PPS scores compared to those who did (Table 1). If healthcare professionals who lack moral sensitivity are less empathetic or more prone to obstetric violence, it may negatively affect students' fear of childbirth.

Another finding was that 53% of the students reported that the births or women's health services they observed changed their views on giving birth, their partner's childbirth, or marriage, with 45.7% of them reporting that their views were negatively affected (Table 1). Sezer et al. [45] stated that midwifery students who witnessed traumatic events during childbirth were more likely to perceive birth as traumatic and to have a fear of childbirth. A study showed that the trauma caused by obstetric violence has both short- and long-term physical and mental effects, negatively impacting women's health and highlighting a serious issue within the healthcare system in Türkiye. Women reported experiencing stress, anxiety, worry, sadness, helplessness, anger, and fear due to obstetric violence during childbirth [50]. In this study, students whose views were changed by births or women's health services had higher and statistically significant pre-birth fear scores (Table 1). Another significant finding was that the predictor of pre-birth fear in students was their perception of obstetric violence (Table 4). Larsson et al. [8] showed that women with a fear of childbirth often had negative experiences in healthcare settings, which could be summarized as disrespectful care and obstetric violence. They also pointed out that the situations women faced in healthcare services could be an underlying cause of their fear of childbirth, and this should be explored further. Women with a fear of childbirth often mentioned previous negative birth experiences or dissatisfaction with intrapartum care [51]. Obstetric violence can lead to trauma related to childbirth and the development of fear of childbirth [52]. In this study, students' exposure to or witnessing obstetric violence may have contributed to an increase in their fear of childbirth.

In this study, a weak relationship was found between students' CF-PPS scores and the subdimensions of PercOV-S, such as “intrapartum mistreatment” and “evidence-based routine practices.” Additionally, a negative and weak relationship was found between ADA scores and “intrapartum mistreatment” (Table 3). Chen et al. [53] identified that professional value mediates the relationship between moral sensitivity and ethical decision-making. Nursing students with higher moral sensitivity were able to integrate professional quality into clinical practice, particularly in terms of respecting patients and protecting patient rights, thereby improving patient-related outcomes [54]. It is stated that moral sensitivity increases nurses' attention to ethical aspects of care quality [23]. As nurses' moral sensitivity increases, the quality of nursing care for patients also improves [17]. In a study by Amiri et al. [47], while no significant relationship was found between nurses' moral sensitivity and patient care quality, an inverse significant relationship was identified between the dimension of ethical conflicts and the dimension of care quality.

This suggests that higher moral sensitivity in nursing students could contribute to enhancing the quality of care they provide. It implies that nurses with a strong moral compass may be more attuned to ethical concerns and thus deliver better patient care. The weak correlations found in this study between students' perceptions of obstetric violence and the fear of childbirth further highlight the need for improved training and awareness in addressing ethical issues, ensuring that future nurses are better equipped to handle such situations.

As providing opportunities for evidence-based thinking and discussion about obstetric violence at universities can have a positive impact on the future of nurses, particularly in maternal and child care, it is crucial to focus on strengthening the professional values and moral sensitivity of nursing students [42]. Raising awareness of obstetric violence and its implications can play a significant role in reducing the perception of obstetric violence, ultimately leading to better healthcare outcomes for both mothers and infants.

In this regard, integrating education on ethical practices and obstetric violence into nursing curricula is essential. By fostering a deeper understanding of these issues, nursing students will be better equipped to recognize and address ethical dilemmas in clinical practice, ensuring that future healthcare professionals are capable of providing compassionate, respectful, and high-quality care in maternity settings. This approach could help prevent the perpetuation of obstetric violence and contribute to a more supportive environment for women during childbirth.

### 4.1. Limitations of the Study

This study has several limitations. First, the cross-sectional design of the research makes it difficult to establish cause-and-effect relationships. Second, since the personal information form and the scales used are based on self-report and contain retrospective information, the accuracy of the results may be influenced by participants' recall difficulties or response biases. Third, the study is limited to nursing undergraduate students, and the findings may not be generalized to individuals from other healthcare disciplines or different educational levels. Given these limitations, the generalizability of the results is limited. However, the strength of this study lies in being the first to examine the relationships between obstetric violence perception, pre-pregnancy fear of childbirth, and moral sensitivity among nursing students. This contributes to filling gaps in the field and providing a foundation for future research.

## 5. Conclusion

This study shows that the average scores of students on pre-pregnancy fear of childbirth, perception of obstetric violence, and moral sensitivity are above the middle level. It was found that students who wish to give birth by cesarean section in the future and those who have witnessed obstetric violence had significantly higher average pre-pregnancy fear of childbirth scores. A weak relationship was found between students' scores for pre-pregnancy fear of childbirth and perception of obstetric violence. Additionally, students who did not think that healthcare professionals were aware of their moral responsibility during childbirth had significantly higher and meaningful pre-pregnancy fear of childbirth scores compared to those who thought so. It was also found that the predictor of students' pre-pregnancy fear of childbirth was their perception of obstetric violence, and there was a negative and weak relationship between moral sensitivity scores and intrapartum mistreatment scores.

### 5.1. Implications for Practices

In order for nursing students' perceptions of obstetric violence, fear of childbirth, and moral sensitivity to develop in a healthier way, ethical values should be emphasized in education programs, and their awareness should be increased on this issue. Providing students with in-depth information on obstetric violence awareness and its effects will help how fear of childbirth affects their professional attitudes.

During clinical practice, nursing students should be provided with feedback and support on the definition of obstetric violence levels and intervention methods, and opportunities should be created and encouraged for students to apply professional ethical values.

It is recommended that studies should be conducted with larger samples in future studies. At the same time, conducting comparative studies with nursing department students from different universities will be useful in determining the effect of education systems on students' ethical decision-making processes. It is recommended that follow-up studies be planned to show how these parameters develop and change during students' professional development processes.

## Figures and Tables

**Table 1:** Distribution of mean scores based on students' characteristics.

Characteristics	Mean ± SD				
Age	21.67 ± 1.37				

	***n***	**%**	**CF-PPS** **Mean ± SD**	**PercOV-S** **Mean ± SD**	**MSQ** **Mean ± SD**

Class					
3. Class	156	49.5	42.96 ± 9.17	88.55 ± 13.45	92.94 ± 18.12
4. Class	159	50.5	44.22 ± 9.17	89.76 ± 13.04	93.31 ± 19.27
Test and *p*	—	—	*t* = 1.218. *p*=0.97	*t* = −0.810. *p*=0.419	MWU = 12,197.5. *p*=0.80
Sex					
Female	251	79.7	45.54 ± 8.25	90.10 ± 12.71	93.30 ± 18.42
Male	64	20.3	36.00 ± 8.69	85.50 ± 14.68	92.46 ± 19.81
Test and *p*	—	—	***t* = 8.162**. **p** < 0.001	***t* = 2.503**. **p**=0.013	MWU = 7636. *p*=0.543
Chose nursing by preference					
Yes	208	66	43.35 ± 9.11	88.56 ± 12.76	93.32 ± 19.70
No	107	34	44.09 ± 9.32	90.33 ± 14.12	92.75 ± 16.61
Test and *p*	—	—	*t* = −0.679. *p*=0.497	*t* = −1.123. *p*=0.262	MWU = 11,040.5. *p*=0.909
Witnessed vaginal birth					
Yes	97	30.8	44.94 ± 9.47	89.16 ± 13.87	92.53 ± 18.00
No	218	69.2	43.00 ± 9.00	89.16 ± 12.98	93.39 ± 19.01
Test and *p*	—	—	*t* = 1.741. *p*=0.083	*t* = −0.003. *p*=0.998	MWU = 10,440.5. *p*=0.859
Heard birth stories					
Positive	114	36.2	41.32 ± 9.17	90.68 ± 12.87	93.61 ± 16.98
Negative	165	52.4	45.86 ± 8.70	88.65 ± 13.33	92.95 ± 19.58
Not having heard	36	11.4	40.47 ± 8.82	86.72 ± 13.82	92.41 ± 20.06
Test and *p*	—	—	*F* = 1.411. *p*=0.056	*F* = 1.010. *p*=0.464	KW = 0.565. *p*=0.754
Future birth preference (self or partner)					
Vaginal birth	220	69.8	42.33 ± 8.85	89.90 ± 12.56	92.69 ± 18.52
Cesarean section	95	30.2	46.54 ± 9.28	87.45 ± 14.63	94.15 ± 19.10
Test and *p*	—	—	***t* = −3.821**. **p** < 0.001	*t* = 1.514. *p*=0.131	MWU = 9914.50. *p*=0.470
Your own birth method					
Vaginal birth	208	66	43.25 ± 8.80	90.24 ± 12.14	92.95 ± 19.37
Cesarean section	107	34	44.27 ± 9.88	87.08 ± 15.00	93.47 ± 17.34
Test and *p*	—	—	*t* = −0.926. *p*=0.355	***t* = 2.013**. **p**=0.045	MWU = 10,940.0. *p*=0.806

Distribution of average scores based on students' characteristics					
Characteristics	*n*	%	CF-PPS Mean ± SD	PercOV-S Mean ± SD	MSQ Mean ± SD
Hearing the term “Obstetric Violence”					
Yes	120	38.1	42.88 ± 10.17	89.74 ± 13.29	91.57 ± 15.65
No	195	61.9	44.04 ± 8.50	88.81 ± 13.23	94.09 ± 20.30
Test and *p*	—	—	*t* = −1.092. *p*=276	*t* = 0.602. *p*=0.548	MWU = 11,299.0. *p*=0.609
Witnessing obstetric violence					
Yes	57	18.1	45.84 ± 9.20	89.61 ± 14.45	91.98 ± 20.24
No	298	81.9	43.10 ± 9.11	89.06 ± 12.99	93.38 ± 18.35
Test and *p*	—	—	***t* = 2.045**. **p**=0.042	*t* = 0.280. *p*=0.779	MWU = 6866.5. *p*=0.434
Need for communication skills of nurses and midwives					
Yes	281	89.2	43.89 ± 9.16	89.50 ± 13.16	93.25 ± 17.94
No	34	10.8	41.20 ± 9.09	86.35 ± 13.79	92.08 ± 24.27
Test and *p*	—	—	*t* = 1.616. *p*=107	*t* = 1.314. *p*=0.190	MWU = 4205.0. *p*=0.254
Believing that healthcare professionals have ethical responsibility during childbirth					
Yes	222	70.5	42.75 ± 9.31	87.86 ± 12.24	93.13 ± 18.61
No	93	29.5	45.63 ± 8.55	92.26 ± 14.98	93.11 ± 18.96
Test and *p*	—	—	***t* = −2.564**. **p**=0.011	*t* = 0.174. *p*=0.007	MWU = 10,249.0. *p*=0.92
Did the births or health services you observed change your view on childbirth/marriage?					
Yes	167	53	46.57 ± 8.27	89.92 ± 13.33	93.25 ± 20.16
No	148	47	40.25 ± 9.01	88.31 ± 13.13	93.00 ± 16.92
Test and *p*	—	—	***t* = 6.491**. **p** < 0.001	*t* = 1.082. *p*=0.28	MWU = 12,077.50. *p*=0.728
If yes, how did it change?					
Positive	25	7.9	40.24 ± 8.12	90.56 ± 11.54	96.80 ± 22.78
Negative	144	45.7	47.59 ± 7.88	89.54 ± 14.16	92.60 ± 19.42
My opinion did not change	146	46.3	40.24 ± 9.01	88.56 ± 12.61	93.02 ± 17.18
Test and *p*	**—**	**—**	*F* = 1.411. *p*=0.056	*F* = 0.347. *p*=0.707	KW = 0.342. *p*=0.559

*Note: F* = ANOVA test; *t*, *t*-test. PercOV-S, Questionnaire for Measuring Perceived Obstetric Violence. Statistically significant values are shown in bold.

Abbreviations: CF-PPS, Childbirth Fear-Prior to Pregnancy Scale; MSQ, Moral Sensitivity Questionnaire; MWU, Mann–Whitney *U* test; SD, standard deviation.

**Table 2:** Pre-pregnancy fear of childbirth, students' obstetric violence perception scale and moral sensitivity questionnaire mean scores.

Scales	Min–max scores on the scale	Min–max values obtained	Mean ± SD	Cronbach's alpha
Childbirth Fear-Prior to Pregnancy Scale	10–60	15–60	43.60 ± 9.17	0.893
Questionnaire for Measuring Perceived Obstetric Violence	27–135	31–120	89.16 ± 13.24	0.844
Intrapartum mistreatment	8–40	10–69	34.33 ± 5.52	0.819
Non-evidence-based routine practices	7–35	7–30	16.38 ± 4.99	0.777
Postpartum mistreatment	5–25	5–25	15.31 ± 3.50	0.545
Accompaniment and consent	3–15	3–15	10.05 ± 2.20	0.328
Intervention without indications	4–20	5–20	12.99 ± 3.33	0.670
Moral Sensitivity Questionnaire	30–210	51–176	93.13 ± 18.68	0.845

**Table 3:** Relationship between students' pre-pregnancy fear of childbirth, perception of obstetric violence, and moral sensitivity.

Scales and sub-dimensions	Values	CF-PPS	PercOV-S	MSQ	PercOV-S—intrapartum mistreatment	PercOV-S—nonevidence-based routine practices	PercOV-S—postpartum mistreatment	PercOV-S—accompaniment and consent	PercOV-S—intervention without indications
CF-PPS	*r*	1	—	—	—	—	—	—	—
	*p*	—	—	—	—	—	—	—	—
PercOV-S	*r*	0.134^*∗*^	1	—	—	—	—	—	—
	*p*	0.018	—	—	—	—	—	—	—
MSQ	*r*	−0.103	−0.030	1	—	—	—	—	—
	*p*	0.068	0.595	—	—	—	—	—	—
PercOV-S— intrapartum mistreatment	*r*	0.154^*∗∗*^	0.688^*∗∗*^	−0.143^*∗*^	1	—	—	—	—
	*p*	0.006	0.000	0.011	—	—	—	—	—
PercOV-S—nonevidence-based routine practices	*r*	0.126^*∗*^	0.646^*∗∗*^	0.039	0.107	1	—	—	—
	*p*	0.026	0.000	0.492	0.057	—	—	—	—
PercOV-S—postpartum mistreatment	*r*	0.007	0.685^*∗∗*^	0.021	0.356^*∗∗*^	0.299^*∗∗*^	1	—	—
	*p*	0.899	0.000	0.717	0.000	0.000	—	—	—
PercOV-S—accompaniment and consent	*r*	0.045	0.583^*∗∗*^	0.039	0.327^*∗∗*^	0.186^*∗∗*^	0.399^*∗∗*^	1	—
	*p*	0.427	0.000	0.488	0.000	0.001	0.000	—	—
PercOV-S—intervention without indications	*r*	0.069	0.734^*∗∗*^	−0.012	0.340^*∗∗*^	0.454^*∗∗*^	0.398^*∗∗*^	0.403^*∗∗*^	1
	*p*	0.224	0.000	0.831	0.000	0.000	0.000	0.000	—

*Note: r*: Pearson correlation coefficient. PercOV-S, Questionnaire for Measuring Perceived Obstetric Violence.

Abbreviations: CF-PPS, Childbirth Fear-Prior to Pregnancy Scale; MSQ, Moral Sensitivity Questionnaire.

*⁣* ^*∗*^ *p*  < 0.05.

*⁣* ^*∗∗*^ *p*  < 0.01.

**Table 4:** Predictors of students' pre-pregnancy fear of childbirth.

Predictors	*B*	SE	*β*	*t*	*p* Value
Constant	35.387	9.020	—	3.923	<0.001
PercOV-S	0.089	0.039	0.129	2.295	**0.022**
MSQ	−0.049	0.027	−0.099	−1.775	0.077
Age	0.222	0.374	0.033	0.593	0.553

*Note: R* = 0.170. *R*^2^ = 0.028. *F* = 3.069. *p*=0.013. PercOV-S, Questionnaire for Measuring Perceived Obstetric Violence. Statistically significant values are shown in bold.

Abbreviation: MSQ, Moral Sensitivity Questionnaire.

## Data Availability

The data that support the findings of this study are available upon request from the corresponding author. The data are not publicly available due to privacy or ethical restrictions.
